# Tramadol and Tramadol+Caffeine Synergism in the Rat Formalin Test Are Mediated by Central Opioid and Serotonergic Mechanisms

**DOI:** 10.1155/2015/686424

**Published:** 2015-06-04

**Authors:** Norma Carrillo-Munguía, Ma. Eva González-Trujano, Miguel Huerta, Xochitl Trujillo, M. Irene Díaz-Reval

**Affiliations:** ^1^Laboratorio “Farmacología del Dolor” del Centro Universitario de Investigaciones Biomédicas, Universidad de Colima, Avenida 25 de Julio 965, 28045 Colima, COL, Mexico; ^2^Laboratorio de Neurofarmacología de Productos Naturales de la Dirección de Investigaciones en Neurociencias, Instituto Nacional de Psiquiatría Ramón de la Fuente Muñiz, Calzada México-Xochimilco 101, 14370 México City, DF, Mexico; ^3^Unidad de Investigación Dr. Enrico Stefani del Centro Universitario de Investigaciones Biomédicas, Universidad de Colima, Avenida 25 de Julio 965, 28045 Colima, COL, Mexico

## Abstract

Different analgesic combinations with caffeine have shown this drug to be capable of increasing the analgesic effect. Many combinations with nonsteroidal anti-inflammatory drugs (NSAIDs) have been carried out, but, in regard to opioids, only combinations with morphine and tramadol have been reported. The antinociceptive synergism mechanism of these combinations is not well understood. The purpose of the present study was to determine the participation of spinal and supraspinal opioidergic and serotonergic systems in the synergic effect of the tramadol+caffeine combination in the rat formalin test. At the supraspinal level, the opioid antagonist, naloxone, completely reversed the effect of the drug combination, whereas ketanserin, a 5-HT_2_ receptor antagonist, inhibited the effect by 60%; however, ondansetron, a 5-HT_3_ receptor antagonist, did not alter the combination effect. When the antagonists were intrathecally administered, there was a significant reduction in all tramadol-caffeine combination effects. With respect to tramadol alone, there was significant participation of the opioid system at the supraspinal level, whereas it was the serotonergic system that participated at the spinal level by means of the two receptors studied. In conclusion, the tramadol+caffeine combination synergically activated the opioid and serotonergic systems at the supraspinal level, as well as at the spinal level, to produce the antinociception.

## 1. Introduction

Analgesic combinations have been used for many years to achieve greater efficacy, enabling the use of lower doses that increase the therapeutic effect (synergism), thus diminishing adverse effects [1]. Caffeine is considered an analgesic adjuvant [2] due to the fact that it has been shown to improve analgesic efficacy when it is coadministered with different analgesics [3–5]. Although many studies suggest that the coadministration of caffeine with different NSAIDs [6, 7] and opioids [8] is able to potentiate the antinociceptive effect, the mechanisms underlying such potentiation are less clearly established.

Tramadol (1RS, 2RS)-2-[(dimethylamino)-methyl]-1-(3-methoxyphenyl)-cyclohexanol is a synthetic opioid analgesic that is used as a racemic mixture of two synergistic enantiomers. It has been called an “atypical” opioid because both an opioid component and a nonopioid component have been demonstrated in its action mechanism [9]. (+)Tramadol is able to bind to the mu receptor, as well as generating serotonin reuptake inhibition and enhancing serotonin release, whereas (−)tramadol preferentially inhibits noradrenaline reuptake [10]. It is widely used for multiple pain conditions, such as postoperative pain, renal colic, dental pain, neuropathic pain, and cancer pain [11]. It has been reported that this drug exhibits good analgesic efficacy and potency comparable to codeine [12].

Caffeine is a methylated xanthine that exerts its effect throughout the antagonism of adenosine A_1_, A_2A_, and A_2B_ receptors [13] at normal human consumption concentrations. Mechanisms such as the inhibition of phosphodiesterase or Ca^2+^ release are activated at high doses of caffeine. In some experimental tests caffeine showed adjuvant and intrinsic analgesic properties and adenosine receptors have been reported to be involved in these effects [14]. Another study reported that serotonergic and noradrenergic systems are related to antinociception in the formalin test [15].

We have reported that caffeine was able to produce a synergic effect when it was coadministered with tramadol. We found ten synergistic combinations and two antagonisms [3]. With these results, we established the synergism that is seen in the tramadol and caffeine combination. However, it is necessary to carry out further analyses to determine if that combination is useful as treatment for pain in humans. Both serotonergic and opioidergic pathways are well recognized participants in pain modulation [16, 17]. In the formalin test, it has been reported that 5-HT_2_ and 5-HT_3_ receptors have a key role in pain modulation at the spinal level [18]. Similar results were observed in the Randall Selitto model [16]. Morphine, when interacting with supraspinal *μ* receptors, activates spinal serotonergic pathways. It has been shown that spinal 5-HT_7_ receptors are involved in the morphine antinociceptive effect, whereas tactile allodynia and thermal hyperalgesia are mediated by spinal 5-HT_3_ receptors [19]. The antihyperalgesic and antinociceptive effects of tramadol are produced in part by spinal 5-HT_7_ receptors [20, 21]. The drugs used in this study have mechanisms that are involved in the descending control pathway at the spinal or supraspinal level. These mechanisms possibly participate in the synergism observed when they are administered in combination. Thus, the purpose of this study was to demonstrate involvement of the serotonergic and opioidergic systems, at both the supraspinal and spinal levels, in the synergic effect of the tramadol plus caffeine combination in the rat formalin test.

## 2. Materials and Methods

### 2.1. Animals

Male Wistar rats weighing 180 to 200 g were used in this study. They were obtained from Harlan, Mexico, and kept in our animal facility. All animals were maintained in a climate-controlled room with a 12 h light/dark cycle. Food was withheld 12 h before the experiments and water was provided* ad libitum*. In all cases, the animals were used only once and were euthanized by cervical dislocation at the end of the experiment. All experimental procedures were conducted in accordance with the recommendations of the Committee for Research and Ethical Issues of the International Association for the Study of Pain [22], the Guidelines on Ethical Standards for the Investigation of Experimental Pain in Animals [23], and the technical specifications for the production, care, and use of laboratory animals of the Mexican Department of Agriculture, Livestock, and Rural Development NOM-062-ZOO-1999 and were carried out according to a protocol approved by the local Animal Ethics Committee. The number of experimental animals was kept at a minimum.

### 2.2. Formalin Test

The formalin model was used to assess antinociception [24]. Each rat was placed in a plexiglass chamber and allowed to move freely for 30 min in order to adapt to the new environment. Mirrors were placed at the back of the chambers to permit full view of the formalin-receiving paw. The rats were then removed and subcutaneously injected with 50 *μ*L of diluted formalin (2%) in the dorsal skin of the hind paw. Recording of the nociceptive behavior was performed in the second phase (begun at 15 min). It was quantified as the number of flinches per minute, every 5 min, up to 60 min after the injection.

### 2.3. Intrathecal Injection

Intrathecal injections (i.t.) were performed as previously described by Yaksh and Rudy [25]. Briefly, the rats were chronically implanted with catheters; they were anesthetized with ketamine and xylazine (75 mg/kg, 12 mg/kg, resp.), were placed in a stereotaxic frame, and underwent surgical procedures that exposed the atlantooccipital membrane by means of an incision in the neck. The membrane was pierced and the tip of a PE-10 polyethylene tube was inserted into the subarachnoid space and carefully advanced 7.5 cm until the tip reached the level of the L4–L6 sections. The incisions were sutured and 8 days were allowed for recovery. The rats were not used if signs of paralysis were observed. The day of the experiment, the animals received the drugs at a volume of 10 *μ*L.

### 2.4. Intracerebroventricular Injection

The rats were deeply anesthetized with a combination of ketamine (75 mg/kg) and xylazine (12 mg/kg) and placed in a stereotaxic frame. Immediately afterwards, a cannula was inserted into the right lateral ventricle (coordinates: lateral 1.8 mm and anteroposterior 0.8 mm) according to the Paxinos and Watson Atlas [26] and then fixed to the skull with dental acrylic. Animals were left to recover for a 48 h period before the experimental session. At the end of the experiments, methylene blue was administered i.c.v. to the rats and they were then anesthetized in an ether chamber. As soon as the animals were in complete anesthesia, they were decapitated; the brain was removed and the zone was located. The day of the experiment, the animals received the drugs at a volume of 4 *μ*L.

### 2.5. Drugs

Tramadol hydrochloride was purchased from Grünenthal Laboratories (Mexico) and ketamine hydrochloride was bought from PISA (Mexico). Xylazine, caffeine, ondansetron, ketanserin, and naloxone were acquired from Sigma-Aldrich (Sigma Chemical Co., MO., USA). Ketanserin was dissolved in 20% DMSO in distilled water and all other substances were dissolved in physiological saline solution.

### 2.6. Study Design

Antagonist drugs were administered i.c.v. and i.t. to the different animal groups. In order to clearly determine the role of the opiate system, the animals were pretreated with the opioid receptor antagonist, naloxone (12 *μ*g/rat). To determine the role of the serotonergic system, the other animal groups received ketanserin, (selective 5-HT_2_ receptor antagonist, 26 *μ*g/rat) and ondansetron (5-HT_3_ receptor antagonist, 16 *μ*g/rat). After a period of 15 min, either tramadol (27.8 mg/kg, s.c.) alone or caffeine (3.2 mg/kg, p.o.) alone and the combination of both drugs (8.8 and 3.2 mg/kg, resp.) were administered to all the groups. These doses were chosen on the basis of previous experiments performed in our laboratory [3]. The antinociceptive effect was measured in the second phase. In the control experiments, saline solution or DMSO (20%) (i.c.v. or i.t.) and tramadol, caffeine, or a combination of both were administered to the other animal groups. The other control groups received naloxone, ketanserin, or ondansetron (i.c.v. or i.t.) and saline solution (s.c. and p.o.). An additional group received saline solution by all routes to assess the magnitude of nociception produced by formalin at 2%. Doses of antagonists were selected on the basis of previous pilot studies under the authors' experimental conditions (data not shown) and data reported by other authors [18, 19]. Tramadol and caffeine were administered 15 min before formalin injection based on pharmacokinetic studies in which these drugs were found in plasma at 15 min [2, 27].

### 2.7. Data Presentation and Statistical Analysis

The nociceptive response was the number of flinches per minute and was scored in the second phase, every 5 min up to 60 min after injection. Drug time courses (TCs) were constructed by graphing the mean number of flinches ± SEM as a time function. The cumulative nociceptive effect was analyzed and determined as area under the curve (AUC) of the TC. AUC was obtained by the trapezoidal rule [28]. Percent of maximum possible effect (%MPE) was regarded as the antinociceptive effect percentage and was calculated from the AUC obtained from the drug group (AUC_D_) and from the control group (AUC_C_) with the following formula:

(1) $$\begin{array}{c} \%MPE=\left[\;\frac{\left({AUC}_{C}-{AUC}_{D}\right)}{{AUC}_{C}}\;\right]\times 100. \end{array}$$

The behavioral test data were presented as the means ± S.E.M. for six experimental observations. An analysis of variance (ANOVA) followed by* post hoc* Tukey's test was carried out for multiple comparisons using SSPS software. A value of *p* < 0.05 was considered statistically significant.

## 3. Results

### 3.1. Synergism

**Figure 1:**
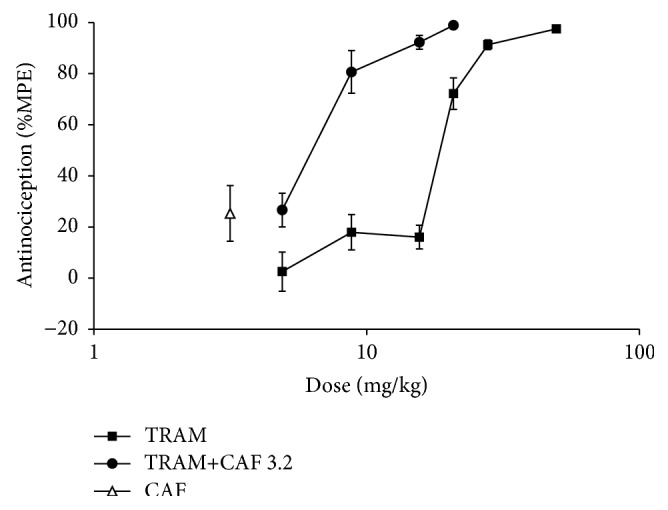
Antinociceptive effects of tramadol (TRAM, s.c.), caffeine (CAF, p.o.), and the combinations of tramadol with caffeine (TRAM+CAF3.2). The doses of tramadol were 4.9, 8.8, 15.6, 20.8, 27.8, and 49.6 mg/kg. The dose of caffeine was 3.2 mg/kg. The combinations were carried out with 4.9 to 20.8 mg/kg of tramadol and 3.2 mg/kg of caffeine. Antinociception was expressed as the percent of maximum possible effect (%MPE) in the second phase of the formalin test. Each point corresponds to the mean ± SEM of six rats.

**Figure 2:**
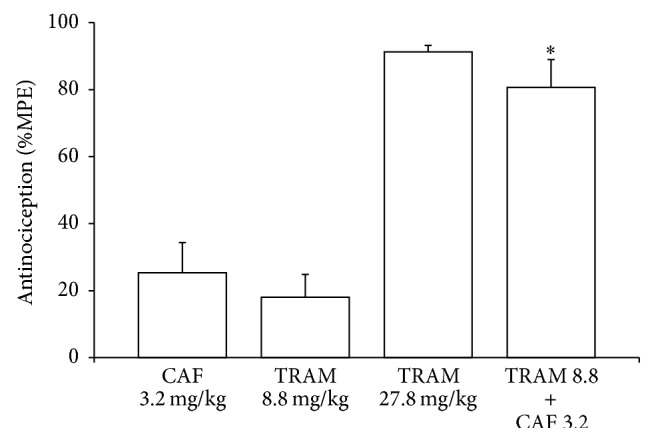
Antinociceptive effects of 3.2 mg/kg of caffeine (CAF), 8.8 and 27.8 mg/kg of tramadol (TRAM), and 8.8 + 3.2 mg/kg of tramadol and caffeine, respectively (TRAM+CAF). Antinociception was expressed as the percent of maximum possible effect (%MPE) in the second phase of the formalin test. Data are presented as mean ± SEM of six rats. (^*∗*^ *p* < 0.05) combination-treated group versus tramadol or caffeine alone.

The dose response curve (DRC) of tramadol presented a dose-dependent effect with an effective dose 50 (ED_50_) of 19.2 mg/kg and a maximum effect of 97.5% that was obtained with a dose of 49.6 mg/kg. When tramadol was administered in combination with 3.2 mg/kg of caffeine, the DRC shifted to the left and there was a significant change in the ED_50_ that resulted in 6.4 mg/kg; maximum effect (98.9%) was obtained with a dose of 20.8 mg/kg (Figure 1). To analyze the mechanisms that participated in the synergism that was produced by the combined administration of tramadol and caffeine, 8.8 mg/kg and 3.2 mg/kg were used, respectively.  Figure 2 shows the antinociceptive effects of the different doses used. Simple tramadol administration at a dose of 8.8 mg/kg presented a percentage effect of 18 ± 6.8% and simple caffeine at a dose of 3.2 mg/kg presented a percentage effect of 25.4 ± 9.8%. However, when both drugs were administered in combination at the doses indicated, the effect increased to 80.7 ± 8.4%. This effect was similar with a tramadol dose that was three times higher (27.8 mg/kg; 91.3 ± 1.9%).

### 3.2. Opioid Mechanism

**Figure 3:**
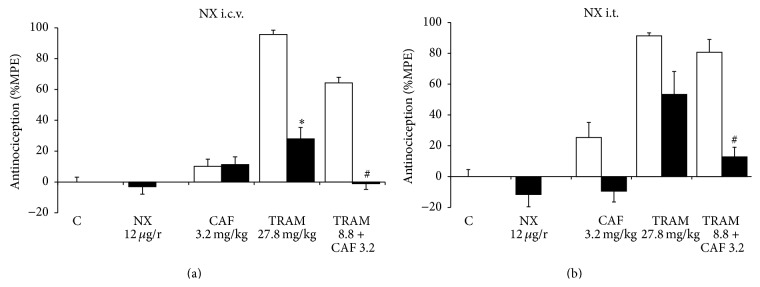
Effect of naloxone (NX) on the antinociceptive effect of caffeine (CAF, p.o.), tramadol (TRAM, s.c.), and tramadol+caffeine (TRAM+CAF). The control group (C) received saline solution by all routes to assess the magnitude of nociception produced by formalin at 2%. The open columns represent the antinociceptive effect of the different treatments with saline solution pretreatment. The dark columns show the effect of NX 15 min before administration of the different treatments. (a) Naloxone was administered i.c.v. (b) Naloxone was administered i.t. Data are presented as mean ± SEM, *n* = 6; ^*∗*^ *p* < 0.05 tramadol versus tramadol with naloxone, ^#^ *p* < 0.05 TRAM+CAF versus TRAM+CAF with naloxone.

The opioid receptor antagonist, naloxone, was used in the analysis of opioid pathway participation. Intracerebroventricular (i.c.v.) injection of naloxone presented no antinociceptive effect. When the animals received pretreatment with naloxone and later were given caffeine, the antinociceptive effect was not altered. On the other hand, the group that was given tramadol after pretreatment with naloxone presented a 70.8% reduction in the antinociceptive effect. This was statistically significant when compared with the group that received just tramadol (*p* < 0.05). The animals that were given naloxone i.c.v. and afterward the combination, did not present an antinociceptive effect. In other words, the opioid antagonist completely inhibited the effect (*p* < 0.05). This suggests opioid mechanism participation (Figure 3(a)).

When opioid antagonist pretreatment was administered by intrathecal injection (i.t.), the effect of both caffeine and tramadol tended to diminish; however, there was no statistically significant difference. These results suggest that there is no important participation by the opioid system at the spinal level for these drugs. However, the combination's antinociceptive effect presented an inhibition percentage of 84.1% when the antagonist was administered (*p* < 0.05), suggesting that the opioid inhibitory pathway participates in the antinociceptive synergism of the combination at the spinal level (Figure 3(b)).

### 3.3. Serotonergic Mechanism

**Figure 4:**
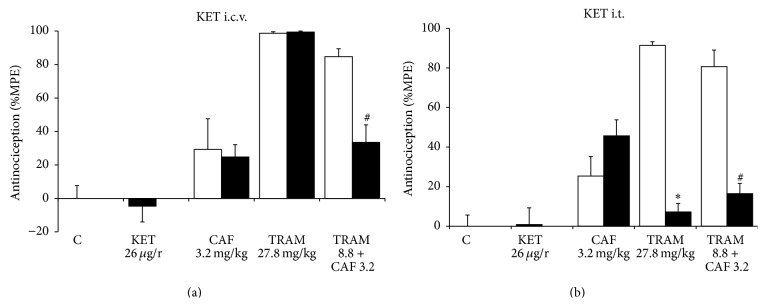
Effect of ketanserin (KET) on the antinociceptive effect of caffeine (CAF, p.o.), tramadol (TRAM, s.c.), and tramadol+caffeine (TRAM+CAF). The control group received saline solution by all routes to assess the magnitude of nociception produced by formalin at 2% (C). The open columns represent the antinociceptive effect of the different treatments with DMSO solution (20%) pretreatment. The dark columns show the effect of KET 15 min before administration of the different treatments. (a) Ketanserin was administered i.c.v. (b) Ketanserin was administered i.t. Data are presented as mean ± SEM, *n* = 6; ^*∗*^ *p* < 0.05 tramadol versus tramadol with ketanserin, ^#^ *p* < 0.05 TRAM+CAF versus TRAM+CAF with ketanserin.

The participation of serotonergic mechanisms in the combination's antinociceptive effect was analyzed using ketanserin, a selective 5-HT_2_ receptor antagonist, and ondansetron, a 5-HT_3_ receptor antagonist, which were administered both i.c.v. and i.t. In the analysis of groups that received pretreatment with ketanserin i.c.v. (Figure 4(a)), there were no alterations in the group treated with caffeine or in the group treated with tramadol, compared with their respective control groups. Nevertheless, in the combination there was a 60.4% reversal of synergism with ketanserin pretreatment (*p* < 0.05).

On the other hand, when ketanserin was administered i.t., it significantly reversed the effect of tramadol (*p* < 0.05). This effect was similar to the control group. With the antagonist pretreatment, the combination's antinociceptive effect was also significantly reversed (*p* < 0.05), obtaining an effect of only 16.5 ± 5.2%, whereas the group that was given just the combination presented an effect of 80.7 ± 8.4% (Figure 4(b)).

**Figure 5:**
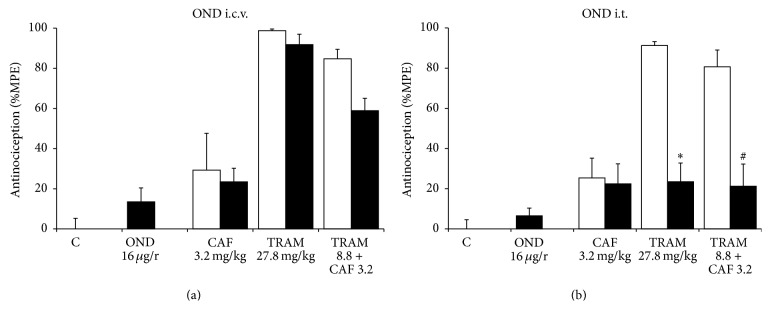
Effect of ondansetron (OND) on the antinociceptive effect of caffeine (CAF, p.o.), tramadol (TRAM, s.c.), and tramadol+caffeine (TRAM+CAF). The control group received saline solution by all routes to assess the magnitude of nociception produced by formalin at 2% (C). The open columns represent the antinociceptive effect of the different treatments with saline solution pretreatment. The dark columns show the effect of OND 15 min before administration of the different treatments. (a) Ondansetron was administered i.c.v. (b) Ondansetron was administered i.t. Data are presented as mean ± SEM, *n* = 6; ^*∗*^ *p* < 0.05 tramadol versus tramadol with ondansetron, ^#^ *p* < 0.05 TRAM+CAF versus TRAM+CAF with ondansetron.

The group receiving ondansetron i.c.v. did not present an antinociceptive effect. Likewise, it did not alter the effect of caffeine or tramadol. On the other hand, when the combination was administered to rats that had previously been treated with the antagonist, there was a slight tendency towards effect reduction (30.5%), compared with the group that received just the combination (Figure 5(a)). These results suggest that the 5-HT_3_ receptor does not participate in the antinociceptive synergism of the combination studied.

Ondansetron administration i.t. did not alter caffeine's antinociceptive effect. Tramadol's antinociceptive effect was importantly reversed in the group pretreated with ondansetron (*p* < 0.05). It only reached an antinociceptive effect of 23.4 ± 9.3% compared with the effect of 91.3 ± 1.9% obtained with tramadol. Interestingly, the combination's effect was significantly reduced (*p* < 0.05). When ondansetron was administered 15 min before, the effect had a reversal of 73.7% (Figure 5(b)).

## 4. Discussion

The combinations of NSAIDs with caffeine have been studied in certain pain models and have shown that caffeine potentiates the analgesic effect. In relation to opioids, the combination of morphine with caffeine has been studied in some pain models [8, 29, 30]; caffeine also potentiated the opioid effect. The present authors reported on the combination of tramadol with caffeine [3]. That study was the basis for analyzing the mechanisms participating in the synergism that presented in the combination.

### 4.1. Central Opioid and Serotonergic Mechanisms of Tramadol

The dorsal horn of the spinal cord is a very important region in regard to nociceptive transmission and modulation [31]. The endogenous opioids, serotonin and noradrenaline, are the principal neurotransmitters that participate in pain modulating descending systems. In the present study, the antagonists were administered with caffeine (3.2 mg/kg) alone and with tramadol (27.8 mg/kg) alone. The dose of caffeine was the one that induced synergism. The caffeine mechanisms were analyzed with that dose because it is well documented that the effect caffeine presents is dose-dependent [14, 29]; thus activated mechanisms are different [15]. The aim of our study was to observe the synergism mechanisms at that dose of caffeine. The effects of both tramadol and caffeine at the indicated doses enabled the inhibition induced by the antagonists to be analyzed. In regard to the groups receiving caffeine in the presence or absence of naloxone, the antagonist did not alter the antinociceptive effect. The results of this study concur with those previously published by other authors that reported that opioid mechanisms did not participate in the antinociceptive effect of caffeine in a formalin model [32].

The analysis of the opioid and serotonergic system carried out in the present study showed that both systems participated in tramadol's effect. It has been extensively reported that tramadol is an atypical opioid analgesic since, in addition to activating opioid receptors, it also inhibits noradrenaline and serotonin reuptake [10, 33]. In the present study, the antinociceptive effect was importantly reversed (70.8%) in the group that was given naloxone i.c.v., suggesting that the most important mechanism for tramadol at the supraspinal level is opioid. This study analyzed supraspinal serotonin participation, and ondansetron or ketanserin administration did not inhibit tramadol's effect. However, there was another participating mechanism indicated by the naloxone results, suggesting possible noradrenergic mechanism participation. The serotonergic mechanism is not ruled out, because the study by Raffa et al. showed that* in vitro* tramadol was capable of inhibiting serotonin reuptake in frontal cortex slices [10]. Another study carried out in rats* in vivo* reported that tramadol increased serotonin levels in the ventral hippocampus [34]. These results suggest that, at the supraspinal level, the serotonergic system in the antinociceptive mechanism of tramadol, evaluated in a formalin model, participates by means of other receptors.

On the other hand, it has been reported that tramadol is capable not only of inhibiting reuptake, but also of inducing serotonin release in the dorsal raphe nucleus [35]. Likewise, the raphe nucleus sends projections to the dorsal horn of the spinal cord, and the direct raphe nucleus stimulation has produced analgesia in some experimental models [36]. The results of our study suggest that there is no opioid system participation at the spinal level in tramadol's antinociceptive effect since the effect tended to decrease in the presence of the opioid antagonist (naloxone), albeit with no significant difference. However, at this level the predominant mechanism was serotonergic by means of the 5-HT_2_ and 5-HT_3_ receptors, since inhibition was practically complete with ketanserin and was 74.3% with ondansetron. The serotonergic mechanism of tramadol has been determined through the systemic administration of antagonists. For example, Oliva et al. reported the participation of the 5-HT_2_ receptor. They determined that ketanserin administered i.p. in the mouse was capable of reversing tramadol's effect, using the formalin model [33]. In addition, in an arthritic pain model, ketanserin (i.p.) also reversed tramadol's effect, and when tramadol was chronically administered, the mRNA of the 5-HT_2_ receptors was increased in the nucleus of raphe magnus (NRM) cells, as well as in the PAG [37]. There are studies using the formalin model that show that the two serotonergic receptors, 5-HT_2_ and 5-HT_3_, participate in the modulation of pain at the spinal level [16, 18]. Based on these pieces of evidence we decided to administer ketanserin and ondansetron locally (i.c.v. and i.t.) and very important 5-HT_2_ receptor participation was observed at the spinal level, but not at the supraspinal level. The role that 5-HT_3_ receptors play in regard to pain is controversial. Reports have stated that these receptors are not involved in the thermal pain inhibition produced by tramadol [20], whereas it was observed that in humans ondansetron inhibited tramadol's analgesic effect in postoperative pain [38]. On the other hand, Dogrul et al. report that the spinal 5-HT_3_ receptor participates in the antiallodynic and antihyperalgesic effect of tramadol, whereas the 5-HT_7_ receptors participate in the antinociceptive effect [19]. A recent study shows that a 5-HT_7_ antagonist reverts the effect of tramadol in the formalin model. The present results show that intrathecal ondansetron administration significantly inhibited tramadol's effect and thus, in the case of inflammatory pain, this receptor probably plays a role in nociceptive transmission inhibition. The results found in the different experimental models suggest that the participating receptors in the antinociceptive effect of tramadol depend on the nociceptive stimulus. It has been proposed that the 5-HT_3_ receptors participate when the nociceptive stimulus induces the release of substance P, as what occurs in the second phase of the formalin model [39]. Thus, in our study, the effects shown with the antagonists suggest that subcutaneous tramadol activates supraspinal *μ* receptors. This activates serotonergic pathways that are projected onto the dorsal horn of the spinal cord, where serotonin is probably released. In this region, serotonin interacts with the 5-HT_2_ and 5-HT_3_ receptors to modulate pain.

### 4.2. Central Opioid Mechanisms and the Tramadol+Caffeine Synergism

The mechanisms participating in the synergism produced by the combination were different with respect to tramadol alone. Opioid participation at the supraspinal level was the most important mechanism both for the effect of tramadol alone and for the combination effect. In both groups, naloxone significantly reversed the antinociceptive effect, but it completely eliminated the effect in the combination. This suggests that the opioid system was activated more by the drug combination than by tramadol alone, which is an interesting fact, given that this mechanism did not participate in caffeine's antinociceptive effect.

One of the mechanisms of tramadol occurs through *μ* receptor interaction [10]. The *μ* receptors are found in important supraspinal nuclei of the descending inhibitory pathway such as PAG, NRM, or the locus coeruleus (LC). The interaction of the opioid system and adenosine in the nociceptive modulation has been reported, but results are controversial. It is well established that A_1_ receptors are found in the dorsal horn of the spinal cord [21]. Some authors have reported that the spinal A_1_ receptors are the ones that participate in the antinociceptive effect of morphine [40], whereas other studies have reported that it is the A_2_ receptor that participates [41]. Nevertheless, with the i.c.v. administration of beta endorphins in the same experimental model, both adenosine receptors participate [41]. A recent study using the formalin model in mice reported that caffeine was capable of reversing the effect of tramadol and it was determined that the spinal A_1_ receptors participated in the analgesic effect [21]. This evidence suggests that the antagonism of the A_1_ receptors is not the mechanism for producing synergism, probably due to the animal species employed. The A_2_ receptors are found mainly in supraspinal sites and it has also been proposed that they are presynaptically located in descending fibers [42]. A pronociceptive role has been attributed to these receptors [43]. Therefore, when caffeine interacts with them, most likely the antinociceptive effect of tramadol increases. More studies are needed to make conclusions in regard to this mechanism.

On the other hand, Borghi et al. demonstrated that the noxious stimulus increased the density of the *μ* receptors in the spinal cord and that the purinergic agonists reduced it in both the spinal cord and the PAG [44]. This evidence, together with the results of our study, suggests that caffeine, a nonselective antagonist of the purinergic receptors, when administered systemically, does not permit the reduction of the *μ* receptors, and therefore a greater density of these receptors can be activated by tramadol. This could explain the inhibition of the effect that naloxone i.t. produces on the combination effect. This effect was not significant when tramadol alone was administered.

### 4.3. Central Serotonergic Mechanisms and the Tramadol+Caffeine Synergism

Serotonin is one of the neurotransmitters that participates in pain modulation. In the antinociceptive synergism caused by the combination at the supraspinal level, the results showed that there was 5-HT_2_ receptor participation, but not 5-HT_3_ receptor participation. The participation of the serotonergic system by means of the 5-HT_2_ receptors is possibly related to the tramadol mechanism, as well as to the caffeine mechanism. It has been reported that tramadol releases serotonin in supraspinal centers [10, 34]. In relation to the antinociceptive effect of caffeine evaluated in the formalin model, it has also been reported that there is serotonergic pathway participation. This is attributed to supraspinal actions [15] that are likely related to catabolism decrease or serotonin release [45]. In these last two studies, high caffeine doses were used. The results of the present study showed no alteration of the antinociception at a caffeine dose of 3.2 mg/kg when serotonergic antagonists were administered. However, when caffeine is administered in combination with tramadol this mechanism would probably be activated, although further studies are needed to be able to confirm this.

Enkephalinergic neurons stimulation in the PAG activates the spinal analgesic systems, including the serotonergic system. The results presented here show that, by means of the receptors studied and at the spinal level, the opioid pathway, as well as the serotonergic pathway, has the same degree of importance in synergism resulting from the combination. Opioid and serotonergic system interaction has also been reported in the dorsal horn of the spinal cord. The 5-HT_2_ and 5-HT_3_ receptors are densely expressed in this region; the role of these receptors in relation to pain is controversial since in some studies they have been seen to participate in antinociception and, in other studies, their participation has been observed in nociception. However, these contradictory results seem to be dependent on the model and animal species used to evaluate pain. Fukushima et al. reported the presence of 5-HT_3_ receptors in GABAergic neurons of the dorsal horn, showing that these receptors are functional [46]. Moreover, another study showed that the activation of the 5-HT_3_ receptors in the dorsal horn increased the concentrations of GABA [47]. 5-HT_3_ receptors were also found in neurons that were not GABAergic and their analysis did not determine whether they were excitatory or inhibitory. Other reports indicate the presence of these receptors in excitatory interneurons [48]. The presence of GABA_B_ receptors has been established in glutamatergic neurons; these receptors inhibit glutamate release, resulting in antinociception [49]. The presence of 5-HT_3_ receptors has been found in enkephalinergic neurons in other studies [50, 51]. Those authors showed the interaction between the GABAergic, serotonergic, and opioidergic systems for modulating pain. Our study showed that intrathecally administered naloxone, as well as ketanserin and ondansetron, was capable of inhibiting the antinociceptive response produced by the tramadol+caffeine combination, suggesting that both drugs, when administered in combination, participate in the modulation of pain at the spinal cord level.

## 5. Conclusions

The antinociceptive synergism of tramadol plus caffeine combination is mediated by opioid and 5-HT_2_ receptors at the supraspinal level, while opioid, 5-HT_2,_ and 5-HT_3_ receptors participate at the spinal level. The antinociceptive effect of tramadol is produced by opioid receptors at the supraspinal level and by 5-HT_2_ and 5-HT_3_ receptors at the spinal level.
